# B4GALT1 deficiency attenuates steatohepatitis by regulating the PPARγ/ACSL4 axis

**DOI:** 10.1097/HC9.0000000000000920

**Published:** 2026-03-20

**Authors:** Youjung Chien, Ruiqi Xia, Da Zhou, Yingjie Ai, Linfeng Wu, Xiaoqing Zeng, Shiyao Chen

**Affiliations:** 1 Department of Gastroenterology and Hepatology, Zhongshan Hospital, Fudan University, Shanghai, China; 2 Department of Gastroenterology, Fuzhou University Affiliated Provincial Hospital, Fuzhou, Fujian, China; 3 Endoscopy Center and Endoscopy Research Institute, Zhongshan Hospital, Fudan University, Shanghai, China; 4 Center of Evidence-Based Medicine, Fudan University, Shanghai, China

**Keywords:** MASLD, lipid peroxidation, hepatocytes, B4GALT1, PPARγ, ACSL4

## Abstract

**Background:**

Lipotoxicity, driven by dysregulated lipid metabolism, is a key initiator of hepatocyte injury in metabolic dysfunction–associated steatotic liver disease (MASLD). β-1,4-galactosyltransferase 1 (B4GALT1), which primarily mediates galactosylation of glycoproteins and glycolipids, is involved in the regulation of plasma lipid composition. Previous studies have implicated aberrant glycosylation in MASLD progression. However, the role and underlying molecular mechanisms of B4GALT1 in MASLD progression remain unclear.

**Methods and Results:**

The protein levels of B4GALT1 were elevated in patients with MASLD as well as in a murine model of MASLD induced by choline-deficient, L–amino acid–defined, high-fat diet (CDAHFD), with a more pronounced increase observed in MASH. Hepatocyte-specific *B4galt1*-knockout mice exhibited significantly attenuated hepatic steatosis and inflammation, but not fibrosis. In addition, hepatic B4GALT1 deficiency suppressed the expression of lipogenic genes, reduced lipid accumulation, and inhibited ferroptosis mediated by lipid peroxidation. Mechanistically, B4GALT1 deficiency impaired the N-glycosylation of peroxisome proliferator-activated receptor gamma (PPARγ), leading to its stabilization. Increased PPARγ protein, in turn, transcriptionally repressed acyl-CoA synthetase long chain family member 4 (ACSL4), thereby mitigating lipid peroxidation. Conversely, PPARγ overexpression in steatotic hepatocytes rescued the pro-ferroptotic phenotype driven by B4GALT1.

**Conclusions:**

Our findings suggest that B4GALT1 plays a crucial role in MASLD progression by targeting lipid peroxidation in hepatocytes via the PPARγ/ACSL4 axis, thus highlighting a potential therapeutic target for MASLD.

## INTRODUCTION

Metabolic dysfunction–associated steatotic liver disease (MASLD), formerly known as nonalcoholic fatty liver disease (NAFLD), is the predominant chronic liver disease worldwide, with a prevalence of approximately 30% in the adult population.^1^ The diagnosis of MASLD is based on histological (biopsy) or imaging evidence of hepatic steatosis along with any one of the cardiometabolic criteria.^2^ MASLD encompasses a spectrum from simple steatosis to metabolic dysfunction–associated steatohepatitis (MASH), an aggressive form characterized by inflammation and fibrosis that drives progression to cirrhosis and hepatocellular carcinoma.^3^ Interplay among genetic polymorphisms,^4^,^5^ dysregulated lipid metabolism,^6^,^7^ oxidative stress,^8^ altered immune responses,^9^ and gut microbiota dysbiosis^10^,^11^ has been proposed to explain the pathogenesis of MASLD. However, there are currently no effective treatments to reverse MASLD, highlighting the need for further research on its underlying mechanisms.

Peroxisome proliferator-activated receptor gamma (PPARγ), a ligand-activated transcription factor of the nuclear hormone receptor superfamily, is a crucial regulator of hepatic lipid metabolism and inflammation.^12^,^13^ In addition to its powerful adipogenesis-promoting effect, PPARγ is involved in improving insulin resistance, inflammation, oxidative stress, endoplasmic reticulum stress, and fibrosis in MASLD.^14^ Previous studies have implicated PPARγ in ferroptosis, which is associated with tumor immunology, traumatic brain injury, and intracerebral hemorrhage.^15^,^16^,^17^ Although PPARγ agonists are reportedly effective for treating MASLD in preclinical studies, the precise role of PPARγ in ferroptosis within MASLD is not well understood.

Ferroptosis is an iron-dependent form of regulated cell death driven by excessive lipid peroxidation.^18^,^19^ Previous studies have reported that ferroptosis is involved in various stages of MASLD.^20^ It contributes to the initiation of inflammation in MASH and continues to play a role as the disease progresses.^21^,^22^,^23^ Ferroptosis in hepatic stellate cells (HSCs) can mitigate liver fibrosis in mice,^24^,^25^ whereas ferroptosis in hepatocytes promotes fibrosis.^26^ Given the pivotal role of lipid metabolism in orchestrating ferroptosis,^27^ ACSL4, a key regulator of fatty acid metabolism, is crucial for inducing lipid peroxidation–driven ferroptosis.^28^,^29^ ACSL4 catalyzes the conversion of polyunsaturated fatty acids (PUFAs) to PUFA-CoA, which are eventually converted to lipid hydroperoxides that cause cellular membrane damage.^30^ Although ACSL4 is indispensable for ferroptosis, its exact function in MASLD progression remains to be elucidated.

Glycosylation, a common and diverse form of protein post-translational modification,^31^ has been implicated in MASLD progression. N-glycosylation of sterol regulatory element**-**binding protein cleavage**-**activating protein (SCAP) exacerbates hepatocyte inflammation and lipid accumulation by enhancing ACSS2-mediated H3K27 acetylation.^32^ In addition, N-glycosylation enhances the transcriptional activity of cAMP-responsive element-binding protein H (CREBH), which regulates the production of peroxisome proliferator-activated receptor α and stearoyl-CoA desaturase-1, leading to reduced lipid deposition and attenuated lipotoxicity.^33^ B4GALT1, an important member of the β-1,4-galactosyltransferase (B4GALT) family, converts galactose to N-acetylglucosamine or other glycan acceptors and is involved in the synthesis of complex-type N-linked oligosaccharides in glycoproteins.^34^ Owing to its important role in N-linked glycosylation, it has recently been implicated in physiological processes^35^,^36^ and various tumor types.^37^,^38^,^39^ In particular, B4GALT1 mutations have been shown to modulate plasma lipid composition.^40^,^41^ Given the link between glycosylation and lipid metabolism, we hypothesized that B4GALT1 plays a pathogenic role in MASLD. In this study, we investigated the function of B4GALT1 in hepatocytes and sought to define the molecular mechanisms by which it contributes to steatohepatitis. The RNA-seq data are available in the GEO database under accession number GSE324244.

## METHODS

### Liver tissue samples of patients with MASLD

Liver samples from patients with MASLD were obtained via percutaneous liver biopsy at Zhongshan Hospital, Fudan University. Two certified hepatic pathologists, blinded to all clinical data, independently evaluated each specimen using the NAFLD activity score (NAS), which was calculated as the sum of steatosis, lobular inflammation, and hepatocellular ballooning. Patients with a NAS≥5 were considered to have MASH. The general characteristics of the human MASLD liver specimens are summarized in Supplemental Table S1-5, Figure S1, http://links.lww.com/HC9/C267. The study protocol was approved by the Institutional Ethics Committee of Zhongshan Hospital, Fudan University (no. B2025-476). The study was conducted in accordance with the Declaration of Helsinki, and the committee waived the requirement for informed consent due to the retrospective use of anonymized archival tissue. A) Research conducted in accordance with the Declarations of Helsinki and Istanbul B) Approved by the Institutional Ethics Committee of Zhongshan Hospital, Fudan University, Approval No: [B2025-476] C) Waiver of informed consent noted due to the retrospective use of anonymized archival tissue, approved by the ethics committee.

### Animal models

Six-week-old male C57BL/6 mice were fed a choline-deficient, L–amino acid–defined, high-fat diet (CDAHFD; A06071302: 60% kcal fat, 0.1% methionine and choline-deficient high-fat; Research Diets) (n=6/group) or control chow diet (D12450J: 10% kcal fat; Research Diets) (n=6/group) for 15 weeks. *B4galt1* ^*flox/flox*^ and *Alb-Cre* mice with a C57BL/6 background were purchased from Shanghai Model Organisms Center. To generate hepatocyte-specific *B4galt1*-knockout (*B4galt1* ^*hep−/−*^) mice, *Alb-Cre* mice were crossed with *B4galt1* ^*^*flox/flox*^*^ mice. Littermate *B4galt1* ^*flox/flox*^ animals generated during the same breeding cycle served as controls. Six- to eight-week-old male *B4galt1* ^*^*flox/flox*^*^ and *B4galt1* ^*hep−/−*^ mice were fed a CDAHFD (n=9–10/group) or a control chow diet (n=8/group) for 15 weeks. Mice were maintained in a specific-pathogen-free facility under a strict 12-hour light–dark cycle at 23 °C and 50%±10% relative humidity, with ad libitum access to food and drinking water. All mice were euthanized after CDAHFD or chow diet feeding for 15 weeks. The animal procedures received prior approval from the Institutional Animal Care and Use Committee of Zhongshan Hospital, Fudan University (IACUC, 2025-153) and were executed in strict accordance with the Guide for the Care and Use of Laboratory Animals.

### Cell culture and treatment

The mouse hepatocyte line AML12 and human embryonic kidney line 293T were provided by Shanghai Institutes for Biological Sciences. 293T cells were maintained in the Dulbecco’s Modified Eagle Medium (DMEM) containing 10% fetal bovine serum and an antibiotic mixture (100 U/mL penicillin and 100 μg/mL streptomycin). AML12 cells were cultured in DMEM/F-12 supplemented with 10% fetal bovine serum, 1× insulin-transferrin-selenium, and 40 ng/mL dexamethasone. To induce lipid deposition, AML12 cells were treated with 0.5 mM free fatty acids (FFA) for 24 hours at a ratio of 2:1 (v/v) of oleic acid: palmitic acid. The cells were fixed and stained with Oil Red O (G1262; Solarbio) to evaluate lipid deposition. AML12 cells were incubated with 5 μM GW9662 (S2915; Selleck) or 10 μM pioglitazone (S2590; Selleck) to inhibit or enhance PPARγ activity, respectively. Ferroptosis inducer RSL3 at 5 μM (S8155; Selleck) or ferroptosis inhibitor Fer-1 at 10 μM (S7243; Selleck) was added to the medium to investigate the role of ferroptosis.

### Biochemical analysis

Serum levels of alanine transaminase (ALT), aspartate transaminase (AST), total triglyceride (TG), total cholesterol (TC), low-density lipoprotein (LDL), and non-esterified fatty acids (NEFA) as well as the TG, TC, LDL, and NEFA levels in murine liver tissues were quantified following standardized manufacturer protocols (ALT: cat. no. C009-2; AST: cat. no. C010-2; TG: cat. no. A110-1; TC: cat. no. A111-1; LDL: cat. no. A113-1; NEFA: cat. no. A042-2; Nanjing Jiancheng).

### Enzyme-linked immunosorbent assays

Plasma TNF-α (EK282; Multi Sciences Biotech) and IL-6 (EK206; Multi Sciences Biotech) concentrations were quantified using mouse enzyme-linked immunosorbent assay (ELISA) kits according to the manufacturer’s instructions.

### Histological analysis

Formalin-fixed paraffin-embedded liver tissues were sectioned at 4 μm thickness for histological assessment using hematoxylin and eosin (H&E) staining, Sirius red staining, and immunohistochemistry (IHC). Frozen liver tissue sections were stained with Oil Red O (G1261; Solarbio). For pathological grading, all liver specimens were scored by 2 experienced pathologists according to NAS: steatosis (0–3), inflammation (0–3), and hepatocyte ballooning (0–2). A total NAS≥5 is indicative of a diagnosis of MASH. Lipid droplets and Sirius red**-**positive areas were quantified with ImageJ software (version 1.8.0; USA) for histological evaluation. Immunohistochemical staining was performed using the proportion or intensity of positive signals.

### Transmission electron microscopy

Cubic liver pieces were dissected and fixed with 2.5% glutaraldehyde in 0.1 M sodium phosphate (pH 7.4) for 24 h at 4 °C. The samples were sent to the Electron Microscopy Core Laboratory, School of Basic Medical Sciences, Fudan University, for subsequent processing. The specimens were then visualized under an electron microscope.

### Iron concentration and lipid peroxidation

The procedures for quantification of iron concentration and lipid peroxidation in liver homogenates and hepatocytes are described in the Supplemental Materials, http://links.lww.com/HC9/C267.

### Cycloheximide chase experiment

At 48 hours after transfection of AML12 cells with *B4galt1* siRNA or *B4galt1*-overexpressing plasmid, FFA intervention was performed for 24 hours. The cells were then treated with 200 μg/mL cycloheximide, harvested, and lysed for western blotting analysis at 0, 3, 6, 9, and 12 hours, respectively.

### Co-immunoprecipitation

Cells were transfected with the indicated plasmids for 48 hours and lysed by sonication in lysis buffer (P0013; Beyotime). For immunoprecipitation, total protein lysates were incubated for 3 hours with corresponding antibodies with gentle shaking at 4 °C, followed by the addition of 30 μL of anti-Flag, anti-c-Myc, or protein A/G magnetic beads overnight. The beads were resuspended in 50 μL 5× loading buffer and boiled for 10–15 minutes. Protein samples were subjected to immunoblotting using anti-B4GALT1 (ab121326; Abcam; and LS‑C368884; LifeSpan BioSciences) or anti-PPARγ (16643-1-AP; Proteintech) antibody.

### Chromatin immunoprecipitation assay

Chromatin immunoprecipitation (ChIP) followed by ChIP-quantitative real-time PCR (ChIP-qPCR) was conducted in AML12 cells using Sonication ChIP Kit (RK20258; ABclonal) and DNA Purification Kit (RK30100; ABclonal) according to the manufacturer’s instructions. Following sonication, cross-linked chromatin samples were then subjected to immunoprecipitation by overnight incubation at 4 °C with either anti-PPARγ antibody (2443; CST) or normal mouse IgG (B900620; Proteintech). Immune complexes were subsequently captured by incubation with protein A/G agarose beads (HY-K0202; MCE) for 3 hours. PPARγ-associated chromatin segments immunoprecipitated from the samples were subjected to qPCR analysis to determine binding enrichment. IgG was used as a negative control. The primer sequence of the PPARγ binding site was as follows: forward, 5′-CACACACACACACACACACA-3ʹ; and reverse, 5′-CAGTCACGTAGCCCAGACAT-3’.

### Statistical analysis

Statistical analyses were performed using GraphPad Prism 9.0 (GraphPad Software, San Diego, CA, USA). Data are presented as mean ± standard error of the mean (SEM) unless otherwise stated. Comparison of the means between 2 groups was performed using the Student *t* test under normality assumptions, or the Mann**-**Whitney *U* test when data distribution deviated from normality. One-way ANOVA or Kruskal**-**Wallis test was used to determine statistical significance for experiments with more than 2 groups, followed by the Tukey post hoc test. Statistical significance was set at *p*<0.05.

## RESULTS

### B4GALT1 is upregulated in patients with MASLD and the murine MASLD model

**FIGURE 1:**
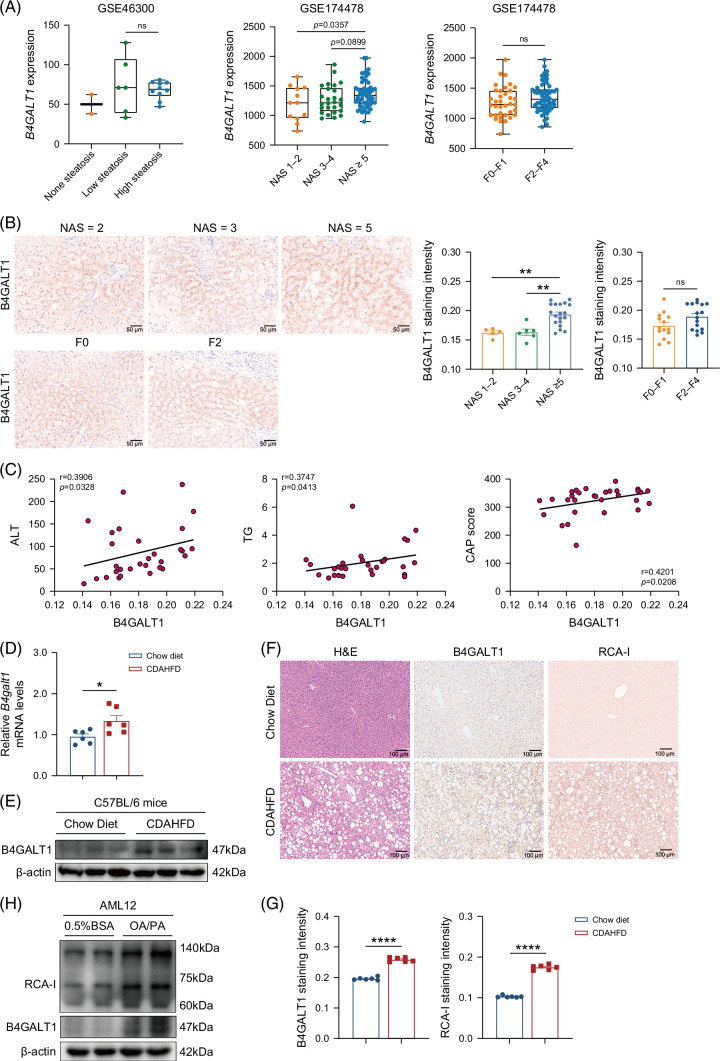
B4GALT1 is upregulated in patients with MASLD and a murine model of MASLD. (A) Relative *B4GALT1* mRNA levels of non-steatosis (n=2), low steatosis (n=6), and high steatosis (n=10) in GSE46300 and of NAS 1–2 (n=12), NAS 3–4 (n=26), NAS≥5 (n=56), F0–F1 (n=36), and F2–F4 (n=58) in GSE174478 datasets. Box and whisker plots display the median (horizontal bar), interquartile range (box limits), and 1.5×interquartile range (whiskers). (B) Representative immunohistochemistry (IHC) images and quantified staining intensity of B4GALT1 classified according to NAS and fibrosis stage in patients with MASLD (NAS 1–2, n=5; NAS 3–4, n=6; NAS≥5, n=19; F0–F1, n=14; F2–F4, n=16). Scale bars, 50 µm. (C) Correlation analysis of serum ALT, TG, and CAP score with B4GALT1 in human MASLD liver samples (n=30). (D) mRNA and (E) protein levels of B4GALT1 in the livers of C57BL/6 mice fed a chow diet or CDAHFD. (F) Representative IHC images of B4GALT1 and RCA-I in liver samples from the control and CDAHFD-fed mice. Scale bars, 100 µm. (G) Quantification of the staining intensity of B4GALT1 and RCA-I in (F) (n=6/group). (H) B4GALT1 and RCA-I protein levels in AML12 cells treated with 0.5% BSA or 0.5 mM FFA. Data are presented as mean ± SEM in (B), (D), and (G). For (A), (B), (D), and (G), significance was determined by the Student *t* test or the ANOVA (*p<0.05, **p<0.01, ****p<0.0001). Abbreviations: B4GALT1, β-1,4-galactosyltransferase 1; BSA, bovine serum albumin; CDAHFD, choline-deficient, L–amino acid–defined, high-fat diet; FFA, free fatty acids; MASLD, metabolic dysfunction–associated steatotic liver disease; NAS, NAFLD activity score; RCA-I, *Ricinus communis* agglutinin I.

To determine whether B4GALT1 is involved in MASLD, we searched hepatic gene expression in a published transcriptome dataset (GSE46300) containing samples from 2 patients with no steatosis, 6 patients with low steatosis, and 10 patients with high steatosis. *B4GALT1* expression was increased in patients with low or high steatosis (Figure 1A). To elucidate *B4GALT1* mRNA levels in MASLD patients with different NAS and fibrosis stages, we retrieved the liver transcriptome data of NAFLD patients (GSE174478). Hepatic tissue *B4GALT1* mRNA levels were significantly elevated in patients with MASH, whereas no significant difference in *B4GALT1* mRNA expression was observed between patients with fibrosis stages F2–F4 versus F0–F1 (Figure 1A). Furthermore, IHC assays revealed a significant upregulation of hepatic B4GALT1 protein in patients with MASH (Figure 1B). However, there was no statistical difference in the levels of B4GALT1 protein between different degrees of fibrosis (Figure 1B). Further correlation analysis showed that hepatic B4GALT1 expression was positively correlated with clinical parameters and total NAS in patients with MASLD (Figure 1C; Supplemental Figures S1A, B, http://links.lww.com/HC9/C267). Similarly, B4GALT1 mRNA and protein levels were increased in the livers of CDAHFD-fed mice for 15 weeks (Figures 1D, E). Moreover, IHC assays of hepatic tissue sections showed elevated *Ricinus communis* agglutinin I (RCA-I) protein levels in MASLD mice (Figures 1F, G). Next, B4GALT1 and RCA-I protein levels were higher in FFA-treated hepatocytes than in the control group (Figure 1H). Altogether, these data demonstrate that B4GALT1 expression is upregulated in both human and murine steatohepatitis.

### Hepatic *B4galt1* loss alleviates fatty degeneration and inflammation in MASLD mice

**FIGURE 2:**
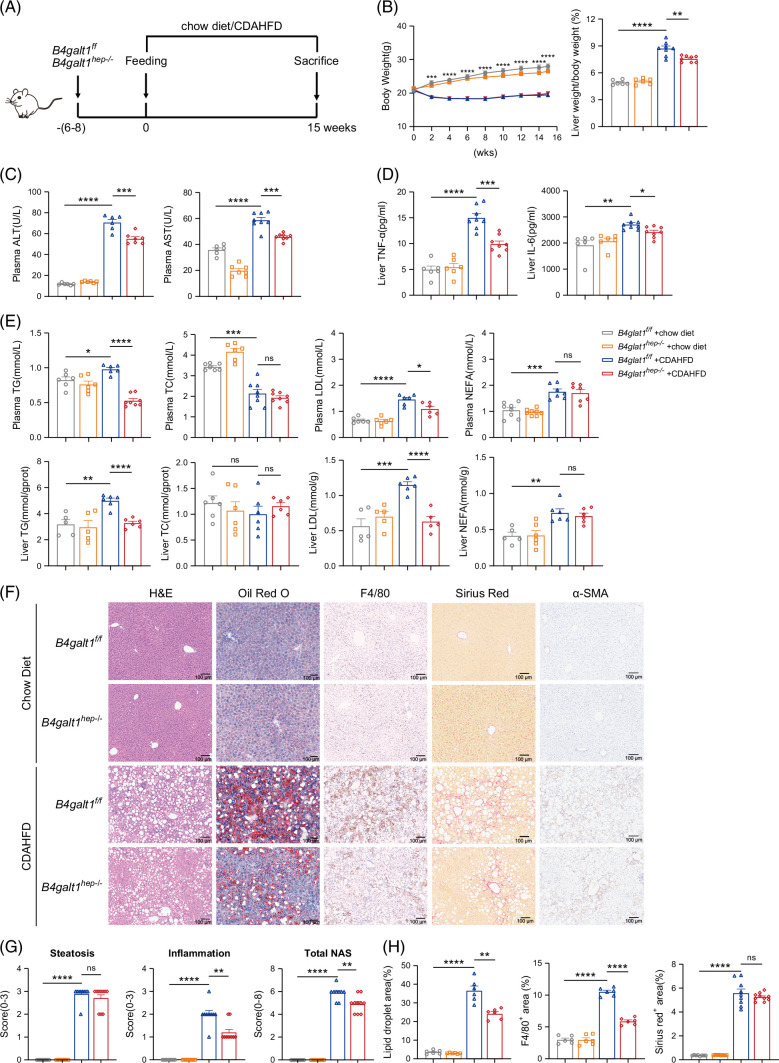
Hepatic loss of B4galt1 alleviates fatty degeneration and inflammation in MASLD mice. (A) Schematic diagram of a murine model fed a chow diet or CDAHFD. (B) Body weight (n=8–10/group) and liver weight/body weight (%) (n=6–8/group) were measured in *B4galt1* ^*flox/flox*^ and *B4galt1* ^*hep−/−*^ mice fed a chow diet or CDAHFD. (C) Serum levels of ALT and AST were measured (n=6–8/group). (D) Levels of TNF-α and IL-6 in the liver tissues were detected using ELISA (n=6–8/group). (E) Serum and hepatic levels of TG, TC, LDL, and NEFA were tested (n=5–8/group). (F) Sections of paraffin-embedded liver tissue were subjected to H&E, Sirius red staining, and immunohistochemical staining for α-SMA and F4/80. Frozen sections were stained with Oil Red O. Scale bars, 100 µm. (G) Steatosis, lobular inflammation scores, and total NAS for each group (n=8–10/group). (H) Quantification of Oil Red O, F4/80, and Sirius red staining. Data are presented as mean ± SEM (*p<0.05, **p<0.01, ***p<0.001, ****p<0.0001; ANOVA). Abbreviations: B4GALT1, β-1,4-galactosyltransferase 1; CDAHFD, choline-deficient, L–amino acid–defined, high-fat diet; ELISA, enzyme-linked immunosorbent assay; H&E, hematoxylin and eosin; MASLD, metabolic dysfunction–associated steatotic liver disease; NAS, NAFLD activity score.

To discern a possible role of B4GALT1 in MASLD, *B4galt1* ^*hep−/−*^ and *B4galt1* ^*flox/flox*^ mice were fed a CDAHFD or a chow diet for 15 weeks (Figure 2A). Starting at 2 weeks, mice fed a CDAHFD showed decreased body weight compared with those fed a chow diet (Figure 2B). The CDAHFD challenge increased the liver-to-body weight ratio, an effect that was reversed by hepatocyte-specific *B4galt1* deletion (Figure 2B). However, the body weight did not differ significantly. ALT, AST, TG, and LDL levels were decreased in the serum and liver homogenates of *B4galt1* ^*hep−/−*^ mice, indicating substantial attenuation of hepatocellular injury and dysregulated lipid metabolism (Figures 2C, E). Analyses of frozen liver sections revealed a significant decrease in fat droplets (Figures 2F, H). Furthermore, *B4galt1* ^*hep−/−*^ mice exhibited lower NAS, reduced macrophage infiltration, and lower TNF-α and IL-6 levels (Figures 2D, F–H). However, histological analyses of liver sections stained with Sirius red and α-SMA revealed no significant changes in fibrosis (Figures 2F, H). Meanwhile, the expression levels of fibrosis-related genes (*Acta2*, *Col1a1*, *Tgfb1*) showed a decreasing trend, although the differences were not statistically significant (Supplemental Figure S2A, http://links.lww.com/HC9/C267). In the liver tissues of the *B4galt1* ^*hep−/−*^ CDAHFD group, the protein levels of fibrosis-related markers α-SMA and MMP9 were not significantly different from those in the *B4galt1* ^*flox/flox*^ CDAHFD group (Supplemental Figure S2B, http://links.lww.com/HC9/C267). In summary, hepatic *B4galt1* knockout alleviated liver steatosis and inflammation in MASLD mice but failed to ameliorate fibrosis.

### Transcriptomic analyses confirmed that B4GALT1 loss mitigates hepatic lipid accumulation

**FIGURE 3:**
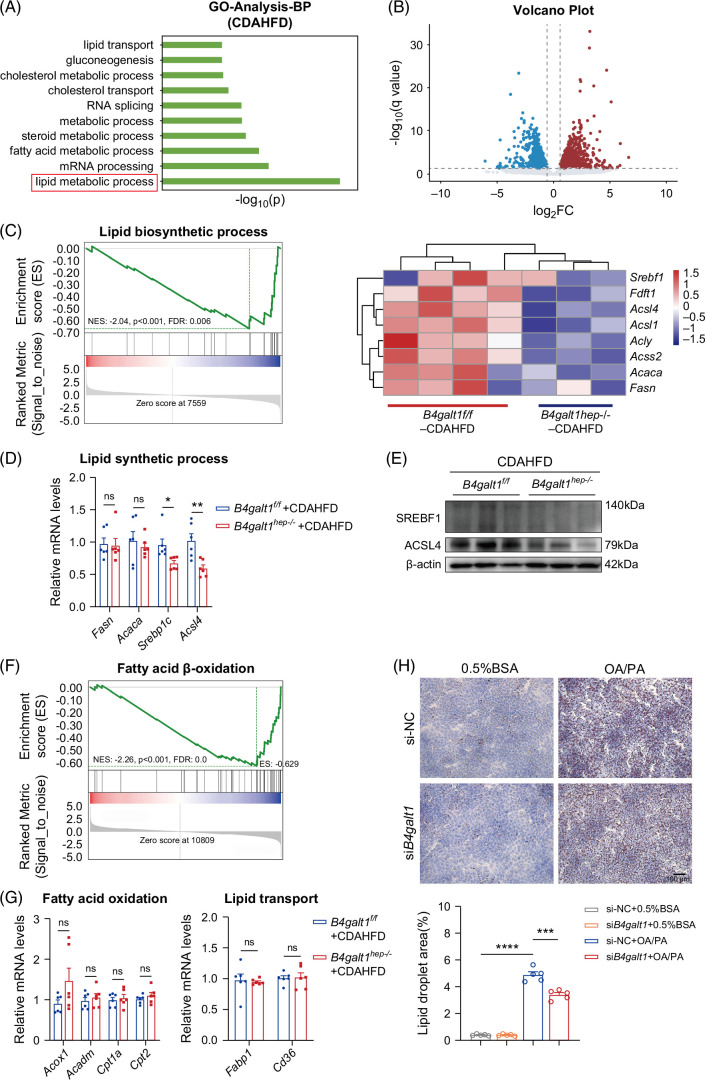
Loss of B4GALT1 mitigates hepatic lipid accumulation according to transcriptome analyses. RNA sequencing was performed on the livers of *B4galt1* ^*flox/flox*^ (n=4) and *B4galt1* ^*hep−/−*^ (n=3) mice fed the CDAHFD. (A) Gene Ontology analysis of RNA-sequencing results in the liver of *B4galt1* ^*flox/flox*^ and *B4galt1* ^*hep−/−*^ mice. (B) Volcano plot depiction of significantly upregulated and downregulated genes. (C) Gene Set Enrichment Analysis plot (left) of enrichment in the lipidsynthetic process signature; heatmap (right) of significantly downregulated target genes. Quantitative PCR (D) and immunoblot (E) analysis of lipidsynthetic-related genes or protein expression in liver samples from CDAHFD-fed mice. (F) Gene Set Enrichment Analysis plot of enrichment in the fatty acid β-oxidation signature. (G) Quantitative PCR analysis of hepatic mRNA expression involved in fatty acid oxidation and lipid transport. (H) Representative images and quantitative analysis of Oil Red O staining in si-NC and si*B4galt1* cells treated with 0.5% BSA or FFA (0.5 mM) for 24 hours. Scale bar, 100 µm. Data are presented as mean ± SEM. For (D) and (G), significance was determined by the Student *t* test. For (H), significance was determined by the ANOVA (*p<0.05, **p<0.01, ***p<0.001, ****p<0.0001). Abbreviations: B4GALT1, β-1,4-galactosyltransferase 1; BSA, bovine serum albumin; CDAHFD, choline-deficient, L–amino acid–defined, high-fat diet; FFA, free fatty acids.

To delineate the role of hepatocyte-specific B4GALT1 loss in MASLD progression, RNA-seq analysis was performed on liver specimens from *B4galt1* ^*hep−/−*^ and *B4galt1* ^*flox/flox*^ mice subjected to a 15-week dietary regimen of standard chow or CDAHFD. Gene Ontology analysis indicated that lipid metabolic processes were remarkably downregulated in *B4galt1* ^*hep−/−*^ mice fed a CDAHFD (Figure 3A). Gene Set Enrichment Analysis (GSEA) revealed that the lipid biosynthetic process was prominently downregulated in *B4galt1* ^*hep−/−*^ mice fed a CDAHFD (Figure 3C). Independent qPCR and immunoblot analyses validated that hepatic deletion of *B4galt1* (*B4galt1* ^*hep−/−*^ mice) elicited marked downregulation of *Srebf1* and *Acsl4*, 2 genes previously identified in the dataset (Figures 3D, E). Besides lipid biosynthesis, lipid deposition is also affected by fatty acid oxidation and transport, and GSEA revealed a coordinated downregulation of the fatty acid β-oxidation gene set (Figure 3F). However, relative gene expression of fatty acid β-oxidation and transport verified by qPCR showed no statistically significant difference (Figure 3G).

To delineate the hepatocyte-intrinsic role of B4galt1 in lipid accumulation, AML12 cells were transfected with B4galt1-targeting small interfering RNA (si*B4galt1*) to achieve efficient gene silencing before steatosis challenge. We found that si*B4galt1* attenuated the accumulation of lipid droplets compared with that in the control group with FFA intervention (Figure 3H). Overall, our findings indicate that hepatic B4GALT1 loss relieves lipid deposition by suppressing lipogenic gene expression.

### B4GALT1 genetic modulation orchestrates lipid–induced hepatocyte ferroptosis in experimental models

**FIGURE 4:**
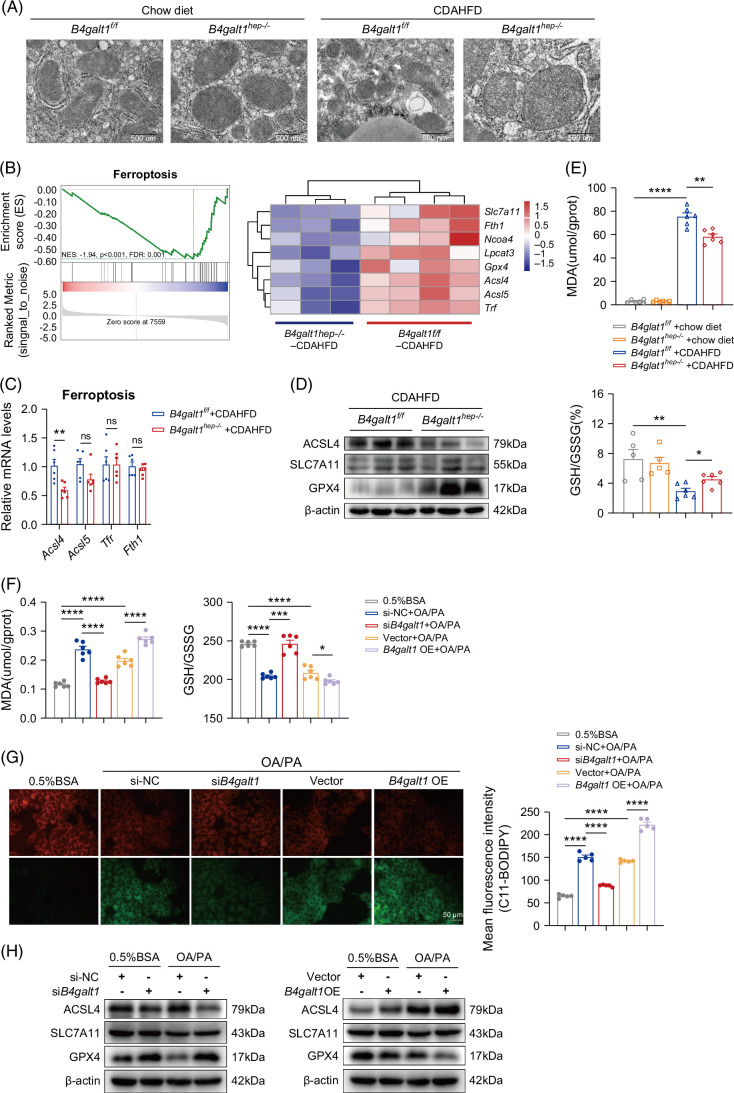
B4GALT1 genetic modulation orchestrates lipid–induced hepatocyte ferroptosis in experimental models. (A) Representative TEM images of the murine model. White arrowheads denote damaged mitochondria (shrunken or inflated mitochondria, reduced number of mitochondrial cristae, and destroyed mitochondrial membrane). Scale bars, 500 nm. (B) Gene Set Enrichment Analysis plot (left) of enrichment in the ferroptosis signature; heatmap (right) of key genes. Quantitative PCR (C) and immunoblot (D) analysis of ferroptosis-related genes or protein expression in liver samples from CDAHFD-fed mice. (E) Level of MDA (n=6/group) and ratio of GSH to GSSG (n=5–6/group) in *B4galt1* ^*flox/flox*^ and *B4galt1* ^*hep−/−*^ mice fed a chow diet or CDAHFD. (F–H) AML12 cells were transfected with si-NC or *B4galt1* siRNA, and vector or *B4galt1* overexpressed (OE) plasmid for 48 hours, and then treated with 0.5% BSA or FFA (0.5 mM) for 24 hours. (F) Level of MDA and ratio of GSH to GSSG (n=6/group). (G) Representative images of C11-BODIPY staining and quantified mean fluorescence intensity of C11-BODIPY images (n=5/group). Scale bar, 50 µm. (H) Immunoblots analysis of ACSL4, SLC7A11, and GPX4. Data are presented as mean ± SEM. For (C), significance was determined by the Student *t* test. For (E), (F), and (G), significance was determined by the ANOVA (*p<0.05, **p<0.01, ***p<0.001, ****p<0.0001). Abbreviations: B4GALT1, β-1,4-galactosyltransferase 1; BSA, bovine serum albumin; CDAHFD, choline-deficient, L–amino acid–defined, high-fat diet; FFA, free fatty acids; GSH, glutathione; GSSG, glutathione disulfide; MDA, malondialdehyde.

Previous studies have suggested that B4GALT1 regulates lipid metabolism in MASLD by inhibiting the expression of lipidsynthetic genes. ACSL4 also plays an important role in ferroptosis mediated by lipid peroxidation. Therefore, we further explored whether B4GALT1 is involved in ferroptosis in MASLD. Transmission electron microscopy revealed that the livers from CDAHFD-fed mice displayed extensive mitochondrial damage (manifested as markedly condensed or swollen organelles, disrupted cristae, and compromised membrane integrity), whereas *B4galt1* deficiency substantially mitigated these ultrastructural defects (Figure 4A). Furthermore, GSEA revealed that ferroptosis was prominently downregulated in *B4galt1* ^*hep−/−*^ mice fed a CDAHFD (Figure 4B). In contrast to the reduced expression of *Acsl4* in *B4galt1* ^*hep−/−*^ mice, no significant differences in the expression of genes related to iron metabolism (*Tfr*, *Fth1*) were observed in *B4galt1* ^*hep−/−*^ mice (Figure 4C). In addition, liver glutathione (GSH), glutathione disulfide (GSSG), malondialdehyde (MDA), and total iron concentrations were measured to evaluate lipid peroxidation and iron overload. Our results indicate that B4GALT1 deletion alleviated CDAHFD-induced lipid peroxidation, as evidenced by GSH/GSSG ratio and MDA levels (Figure 4E). Next, hepatic total iron concentrations were elevated following CDAHFD treatment, although the difference in iron content between *B4galt1* ^*hep−/−*^ and *B4galt1* ^*flox/flox*^ mice was not statistically significant under CDAHFD exposure (Supplemental Figure S3A, http://links.lww.com/HC9/C267).

To further examine the effect of B4GALT1 on ferroptosis in MASLD in vitro, we overexpressed *B4galt1* (*B4galt1* OE) and knocked down *B4galt1* (si*B4galt1*) in mouse AML12 hepatocytes as described previously. In AML12 hepatocytes, FFA overload evoked lipid peroxidation, as evidenced by decreased GSH/GSSG, elevated MDA, and intensified C11-BODIPY fluorescence; these disturbances were reversed upon *B4galt1* knockdown (si*B4galt1*) (Figures 4F, G). FFA exposure differentially modulated ferroptosis regulators with significant downregulation of GPX4 and SLC7A11 and upregulation of ACSL4. Ablation of *B4galt1* downregulated ACSL4 and upregulated GPX4 (Figure 4H). As expected, *B4galt1* overexpression aggravated hepatocyte ferroptosis (downregulating GPX4 and upregulating ACSL4) with severe lipid peroxidation (GSH/GSSG, MDA, and C11-BODIPY fluorescence intensity). To discern the effect of FFA challenge on iron metabolism, intracellular Fe^2+^ levels in hepatocytes were examined using the FerroOrange fluorescent probe. However, the results demonstrated no significant alterations in Fe^2+^ concentrations despite *B4galt1* mRNA level variations (Supplemental Figures S3B, C, http://links.lww.com/HC9/C267).

To decipher whether the protective effect of *B4galt1* silencing against FFA-evoked steatosis was mediated through ferroptosis, AML12 hepatocytes were treated with the ferroptosis inducer RSL3 (5 μM, 24 h) following *B4galt1* knockdown. Inducing ferroptosis with RSL3 reversed the protective effects of *B4galt1* knockdown, restoring high levels of lipid peroxidation in FFA-treated AML12 cells (Supplemental Figures S4A–D, http://links.lww.com/HC9/C267). Conversely, the ferroptosis-specific inhibitor Fer-1 significantly alleviated the aggravated lipid peroxidation evoked by *B4galt1* overexpression in steatotic hepatocytes (Supplemental Figures S5A–D, http://links.lww.com/HC9/C267). Taken together, these results support the protective role of B4GALT1 deficiency against high-fat–induced hepatocyte lipid peroxidation.

### B4GALT1 deficiency inhibits lipid peroxidation through a PPARγ/ACSL4**-**dependent mechanism

**FIGURE 5:**
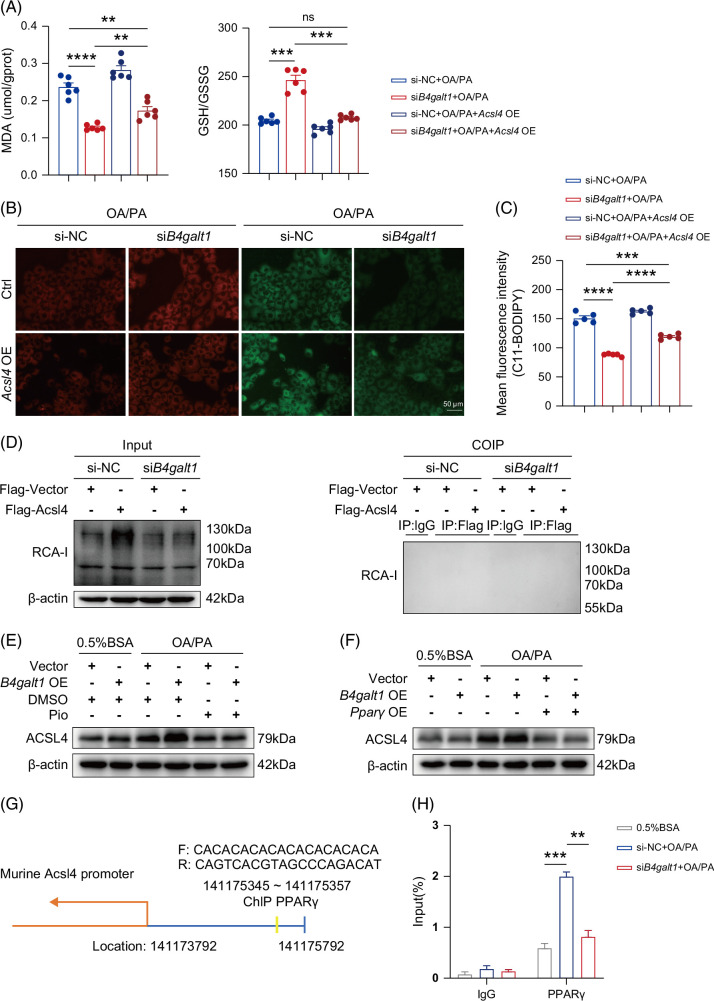
B4GALT1 deficiency suppresses lipid peroxidation via a PPARγ/ACSL4-dependent mechanism. (A–C) AML12 cells were co-transfected with si-NC or *B4galt1* siRNA combined with vector or *Acsl4* overexpressed (OE) plasmid for 48 hours and then exposed to FFA (0.5 mM) for 24 hours. (A) Level of MDA and ratio of GSH to GSSG (n=6/group). (B) Representative images of C11-BODIPY staining. Scale bar, 50 µm. (C) Quantified mean fluorescence intensity of C11-BODIPY images (n=5/group). (D) Immunoblot analysis and ACSL4 immunoprecipitation analysis evaluating RCA-I in AML12 cells. (E) Immunoblot analysis of ACSL4 in AML12 cells transfected with vector or *B4galt1* OE for 48 hours prior to exposure to 0.5% BSA or FFA together with DMSO or pioglitazone (10 μm) for 24 hours. (F) Immunoblot analysis of ACSL4 in AML12 cells co-transfected with vector or *B4galt1* OE combined with *Acsl4* OE plasmid for 48 hours prior to exposure to 0.5% BSA or FFA for 24 hours. (G) Prediction of PPARγ binding sites to the *Acsl4* promoter in the Jaspar database and the corresponding primer sequences. (H) Chromatin immunoprecipitation assays were performed in AML12 cells transfected with si-NC or *B4galt1* siRNA prior to exposure to 0.5% BSA or FFA using antibodies against PPARγ and IgG. Data are presented as mean ± SEM. For (A), (C), and (H), significance was determined by the ANOVA (**p<0.01, ***p<0.001, ****p<0.0001). Abbreviations: ACSL4, acyl-CoA synthetase long chain family member 4; B4GALT1, β-1,4-galactosyltransferase 1; BSA, bovine serum albumin; FFA, free fatty acids; PPARγ, peroxisome proliferator-activated receptor gamma; RCA-I, *Ricinus communis* agglutinin I.

Since transcriptome GSEA of liver tissues showed that ACSL4 is associated with ferroptosis and fatty acid metabolism, we hypothesized that B4GALT1 affects the lipid peroxidation level of hepatocytes via ACSL4. *Acsl4* was overexpressed in hepatocytes with *B4galt1* knockdown to verify the above hypothesis. Lipid reactive oxygen species (ROS) and MDA levels decreased in FFA-induced AML12 cells after *B4galt1* knockdown, whereas *Acsl4* overexpression reverted lipid ROS and MDA levels in cells of the si*B4galt1* group (Figures 5A–C). These data revealed that Acsl4 overexpression partially reversed the hepatoprotective effects of B4galt1 deficiency against FFA-induced lipid ROS.

The activity and function of ACSL4 are regulated at the transcriptional, post-transcriptional, and post-translational levels.^29^ Since B4GALT1 exerts its biological functions primarily by mediating N-glycosylation, we investigated whether it regulates the expression of ACSL4 through N-glycosylation. To study the changes in the β-1,4-galactosylation level of ACSL4 under FFA intervention, an immunoprecipitation experiment of ACSL4 was carried out in AML12 hepatocytes, and the β-1,4-galactosylation level was detected by RCA-I. The results showed that β-1,4-galactosylation of ACSL4 was not detected, suggesting an indirect role for B4GALT1 in regulating ACSL4 (Figure 5D).

Previous studies demonstrated that activation of PPARγ could inhibit the expression and activity of ACSL4, and alleviate radiation-induced intestinal ferroptosis by suppressing ACSL4.^28^,^42^ The *ACSL4* gene contains numerous transcription factorbinding sites including PPAR.^29^ Considering the aforementioned studies, we speculated that B4GALT1 regulates the transcriptional level of ACSL4 through PPARγ, thereby influencing lipid peroxidation in hepatocytes. Western blot analysis revealed that pioglitazone (PPARγ agonist) or *Pparγ* overexpression reversed the changes in ACSL4 protein levels caused by *B4galt1* overexpression under high-fat conditions (Figures 5E, F). This finding denoted that PPARγ might be involved in the regulation of ACSL4 expression by B4GALT1 in FFA-induced hepatocytes.

Due to the role of PPARγ as a transcription factor, we confirmed the potential interaction between PPARγ and the *ACSL4* gene promoter regions in human and mouse genomes using the UCSC Genome Browser. Furthermore, we identified potential PPARγ binding sites within the *Acsl4* promoter region using the JASPAR database (Figure 5G). ChIP-qPCR analysis of hepatocytes indicated that FFA promoted the interaction between PPARγ and the promoter region of the *Acsl4* gene, whereas *B4galt1* knockdown dampened this interaction (Figure 5H).

Taken together, these data demonstrate that B4GALT1 modulated the transcriptional level of ACSL4 via PPARγ, thereby affecting FFA-induced lipid peroxidation levels in hepatocytes.

### B4GALT1 interacts with PPARγ and regulates the protein stability of PPARγ through N-glycosylation to affect lipid peroxidation in MASLD

**FIGURE 6:**
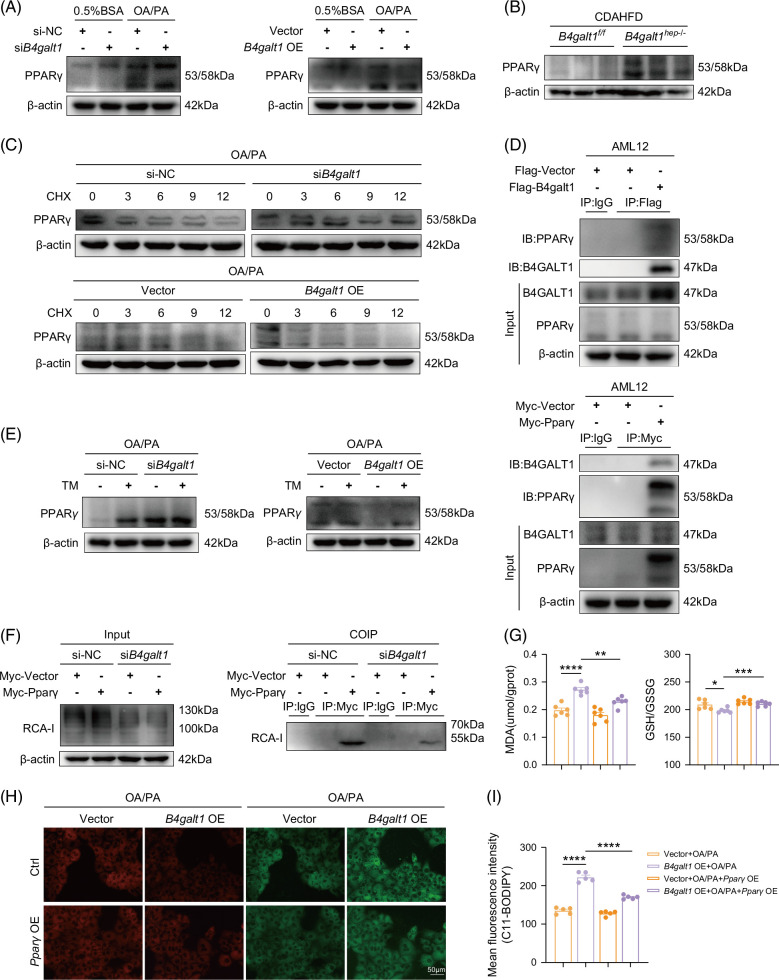
B4GALT1 interacts with PPARγ and regulates the protein stability of PPARγ through N-glycosylation. (A) Immunoblot analysis of PPARγ levels in AML12 cells transfected with *B4galt1* siRNA versus si-NC (left) and in AML12 cells transfected with *B4galt1*-overexpressing (OE) versus vector (right) exposure to either 0.5% BSA or FFA. (B) Immunoblot analysis of PPARγ levels in liver samples taken from *B4galt1* ^*flox/flox*^ and *B4galt1* ^*hep−/−*^ mice fed CDAHFD (n=3/group). (C) Cycloheximide chase assay of PPARγ expression in AML12 cells transfected with *B4galt1* siRNA versus si-NC (top) and in *B4galt1*-overexpressing AML12 cells versus empty vector**-**transfected cells (bottom). (D) Co-immunoprecipitation analysis evaluating the interaction between B4GALT1 and PPARγ in AML12 cells using exogenous Flag-B4galt1 or Myc-Pparγ, Flag-vector, or Myc-vector as a control. (E) Immunoblot analysis of PPARγ levels in *B4galt1*-knockdown AML12 cells, *B4galt1*-overexpressing AML12 cells, and their corresponding si-NC/vector cells after treatment with tunicamycin (TM) and FFA. (F) Immunoblot and PPARγ immunoprecipitation analyses of RCA-I levels in AML12 cells transfected with si-NC or *B4galt* *1* siRNA in combination with Myc-vector or Myc-Pparγ plasmid. (G–I) AML12 cells were co-transfected with vector or *B4galt1* OE in combination with *Acsl4* OE plasmid for 48 hours and then exposed to FFA for 24 hours. (G) Levels of MDA and the ratio of GSH to GSSG (n=6/group). (H) Representative images of C11-BODIPY staining. Scale bar, 50 µm. (I) Quantified mean fluorescence intensity of C11-BODIPY images (n=5/group). Data are presented as mean ± SEM (*p<0.05, **p<0.01, ***p<0.001, ****p<0.0001; ANOVA). Abbreviations: B4GALT1, β-1,4-galactosyltransferase 1; BSA, bovine serum albumin; FFA, free fatty acids; PPARγ, peroxisome proliferator-activated receptor gamma; RCA-I, *Ricinus communis* agglutinin I; TM, tunicamycin.

Previous results suggested that B4GALT1 modulates ACSL4 expression via PPARγ under high-fat conditions. However, the mechanism underlying B4GALT1-mediated regulation of PPARγ remains unclear. Thus, we analyzed PPARγ expression in *B4galt1*-overexpressing cells or knockdown cells in the presence or absence of FFA treatment. *B4galt1* overexpression suppressed FFA-induced PPARγ upregulation in AML12 cells, whereas *B4galt1* knockdown enhanced PPARγ expression (Figure 6A). Otherwise, western blot analysis revealed that PPARγ was significantly upregulated in the liver of *B4galt1* ^*hep−/−*^ mice (Figure 6B). Thus, B4galt1 deficiency upregulated PPARγ expression in the MASLD model. We then performed a co-immunoprecipitation experiment with B4GALT1 or PPARγ antibody using AML12/293T cells, which indicated that B4GALT1 interacted with PPARγ in AML12/293T cells (Figure 6D; Supplemental Figure S6A, http://links.lww.com/HC9/C267). Furthermore, cycloheximide chase assays indicated that *B4galt1* knockdown significantly enhanced the protein stability of PPARγ, whereas *B4galt1* overexpression reduced PPARγ protein stability in FFA-treated AML12 cells (Figure 6C). Since B4GALT1 has been implicated in N-linked glycosylation, we investigated whether B4GALT1 regulates PPARγ protein stability via N-linked glycosylation. *B4galt1*-knockdown or *B4galt1*-overexpressing AML12 cells were co-treated with N-linked glycosylation inhibitor tunicamycin and FFA. Combined with tunicamycin treatment, the expression level of PPARγ protein in AML12 hepatocytes was higher than that in the FFA alone group. Strikingly, co-administration with tunicamycin and FFA significantly upregulated PPARγ expression in *B4galt1*-overexpressing AML12 cells compared with the cells treated with FFA alone (Figure 6E). In addition, in AML12 hepatocytes subjected to high-fat intervention, immunoprecipitation of PPARγ was performed and β-1,4-galactosylation levels were detected using RCA-I, to investigate the impact of B4GALT1 on the β-1,4-galactosylation levels of PPARγ. The β-1,4-galactosylation levels of PPARγ in *B4galt1*-knockdown hepatocytes treated with FFA were reduced compared with the control group (Figure 6F). Overall, these findings suggest that B4GALT1 interacts with PPARγ and regulates the stability of PPARγ protein through N-glycosylation in the presence of high fat.

To examine the role of PPARγ in B4GALT1-mediated ferroptosis in MASLD, we overexpressed *Pparγ* in FFA-induced *B4galt1-*overexpressing AML12 cells and then tested its effect on lipid peroxidation. Notably, *Pparγ* overexpression reversed the changes in MDA levels, GSH/GSSG ratio, and fluorescence intensity of the C11-BODIPY probe caused by *B4galt1* overexpression (Figures 6G–I). Altogether, our data show that PPARγ is involved in the regulation of lipid peroxidation in B4GALT1-mediated MASLD.

## DISCUSSION

Here, we identified glycosyltransferase B4GALT1 as a novel driver of MASLD progression. Hepatic expression levels of B4GALT1 were significantly elevated in both clinical MASLD patients and experimental murine models. Furthermore, hepatocyte-specific deletion of *B4galt1* markedly attenuated hepatic steatosis and suppressed inflammatory responses in the CDAHFD-fed murine model. Mechanistically, B4GALT1 interacted with PPARγ and promoted its degradation via N-glycosylation, resulting in transcriptional upregulation of its target ACSL4 and consequent aggravation of lipid peroxidation in MASLD.

Although glycosylation has been proposed to affect MASLD progression, its role remains inconsistent. N-glycosylation of SCAP exacerbates hepatocyte inflammation and lipid accumulation,^32^ whereas that of CREBH alleviates lipid deposition and lipotoxicity.^33^ However, the cellular role and clinical significance of galactosyltransferases in MASLD remain unclear. Our study provided clinical evidence through IHC analysis that upregulated B4GALT1 expression in patients with MASLD, particularly in those with MASH. Importantly, liver-specific conditional knockout of *B4galt1* significantly alleviated hepatic steatosis induced by a high-fat diet. Moreover, deletion of *B4galt1* in the liver attenuated hepatic inflammation in response to a high-fat diet, whereas no significant changes were observed in liver fibrosis. Our findings suggest that B4GALT1 is a key mediator of the early stages of disease, particularly the transition from simple steatosis to steatohepatitis. Alternatively, hepatocyte-specific B4GALT1 appears to exert only a modest influence on fibrogenesis, whereas its paracrine or cell-autonomous functions in non-parenchymal populations, particularly activated HSCs and Kupffer cells, may be more critical for extracellular matrix deposition.^43^ Diverse cell-specific MASLD models need to be further employed to dissect the spatiotemporal roles of B4GALT1 within the hepatic microenvironment. Of note, previous research suggested that low expression of B4GALT1 promoted liver cancer.^44^ Collectively, our findings underscore that B4GALT1 exerts context-dependent and substrate-diverse biological functions across different disease backgrounds.

Lipotoxic lipids serve as initiating factors for hepatocyte injury and fibrosis during MASLD pathogenesis.^45^ Fatty acid metabolism involves de novo lipogenesis, lipolysis within adipose tissue, mitochondrial oxidation, and lipid transport and uptake.^46^ In this study, genes involved in lipid synthesis, such as *Srebf1* and *Acsl4*, were decreased in *B4galt1* ^*hep−/−*^ mice, while no significant changes were observed in genes associated with fatty acid oxidation and lipid transport, suggesting that B4GALT1 deficiency alleviates hepatic lipid deposition by suppressing lipid synthesis.

Ferroptosis is a form of iron-dependent cell death characterized by high levels of lipid peroxidation.^18^,^19^ Emerging evidence has demonstrated the role of ferroptosis in the different stages of MASLD.^20^ Ferroptosis contributes to the initiation of inflammation in MASH, as well as ferroptosis-related inhibitors or inducers influence its progression.^21^,^22^,^23^ Moreover, during the hepatic fibrosis stage, ferroptosis occurring in different cell types has distinct effects on prognosis.^24^,^25^,^26^ Transmission electron microscopy of liver tissues and hepatic transcriptomics revealed downregulation of ferroptosis in the livers of *B4galt1* ^*hep−/−*^ mice. In addition, decreased lipid peroxidation was observed in the livers of *B4galt1* ^*hep−/−*^ mice, whereas no significant changes were detected in total iron concentration. These findings indicate that B4GALT1 promotes ferroptosis primarily by increasing the availability of lipid substrates for peroxidation (via ACSL4), rather than by altering total cellular iron content. Moreover, the absence of significant changes in intracellular Fe^2+^ levels (Supplemental Figures S3B, C, http://links.lww.com/HC9/C267) indicates that B4GALT1 likely remodels the local labile iron pool (LIP) within specific organelles.^47^ Collectively, we conclude that hepatocyte-specific B4GALT1 deletion mitigates lipidperoxidation-mediated ferroptosis in experimental models of MASLD.

PPARγ, a member of the nuclear receptor superfamily and ligand-activated transcription factor, reportedly regulates lipogenesis, inflammation, oxidative stress, and fibrosis in MASLD.^12^,^14^ Importantly, transcriptomic mining of HFD- and GAN-fed murine MASLD datasets revealed a positive correlation between B4galt1 expression and PPARγ target genes (Supplemental Figures S7A–C, http://links.lww.com/HC9/C267). Moreover, B4GALT1 deletion reduced *Pparγ* mRNA levels (Supplemental Figures S8A–C, http://links.lww.com/HC9/C267). Nevertheless, B4GALT1 downregulated PPARγ expression at the protein level, indicating that its primary impact on PPARγ is exerted through post-translational regulation. Since B4GALT1 has the capacity to modulate glycoprotein stability by N-linked glycosylation,^48^,^49^ we examined the effect of B4GALT1 on PPARγ protein stability. Co-immunoprecipitation and N-linked glycosylation inhibition studies revealed that B4GALT1 interacted with and destabilized PPARγ protein via N-linked glycosylation. Furthermore, immunoprecipitation assays of PPARγ revealed that B4GALT1 regulated the β-1,4-galactosylation level of PPARγ in steatotic hepatocytes. Functional studies indicated that PPARγ overexpression suppressed the elevated lipid peroxidation levels induced by B4GALT1, suggesting its involvement in the regulation of lipid peroxidation mediated by B4GALT1 in MASLD.

In addition to being a key regulator of fatty acid metabolism, ACSL4 plays an important role in lipid peroxidation-driven ferroptosis.^28^,^29^ The activity and function of ACSL4 are regulated at multiple levels;^29^ however, this study did not identify any N-glycosylation modifications of ACSL4. PPARγ activation has been proposed to suppress the expression and activity of ACSL4 and attenuate radiation-induced intestinal ferroptosis by inhibiting ACSL4.^42^,^50^ *ACSL4* gene contains numerous transcription factor-binding sites, including PPAR. In steatotic hepatocytes, PPARγ agonists or PPARγ overexpression significantly reduced ACSL4 expression. Analysis of the *ACSL4* gene promoter sequence revealed potential PPARγbinding sites. Importantly, the ChIP assay indicated decreased PPARγ binding to the *Acsl4* promoter following *B4galt1* knockdown in steatotic hepatocytes. In summary, PPARγ directly binds to the *ACSL4* promoter and represses ACSL4 transcriptional expression; thus, we propose that B4GALT1 regulates ACSL4 expression at the transcriptional level via PPARγ in steatotic hepatocytes.

Several limitations still exist in the present study. First, the CDAHFD-induced MASLD model employed herein, characterized by pronounced weight loss, contrasts with the obese phenotype typical of human MASH. Future studies should corroborate B4GALT1 function in metabolically congruent models. Second, our study illustrated B4GALT1 role in the regulation of hepatocellular lipid peroxidation but did not fully investigate its effects on ferritinophagy or iron transport proteins within the LIP. In addition, the specific contribution of PPARγ to B4GALT1-mediated regulation of MASLD warrants further in vivo validation. Finally, the specific N-glycosylation sites on PPARγ remain unidentified, and their functional impact awaits validation by mass spectrometry and site-directed mutagenesis.

In conclusion, our findings uncover a novel regulatory mechanism between B4GALT1 and PPARγ/ACSL4 signaling pathway and highlight that targeting B4GALT1-mediated N-glycosylation is a promising therapeutic strategy for MASLD.

## Supplementary Material
