# Supplementary material for: B4GALT1 deficiency attenuates steatohepatitis by regulating the PPARγ/ACSL4 axis
Source: Hepatol Commun. 2026 Mar 20;10(4):e0920. doi: 10.1097/HC9.0000000000000920 (PMC13004210; doi:10.1097/HC9.0000000000000920)
Supplement: Supplementary file 1 [file hc9-10-e0920-s001.pdf]

Manuscript ID: HEP4-25-1318

B4GALT1 deficiency attenuates steatohepatitis by regulating the PPARγ/ACSL4 axis

Youjung Chien[1†], Ruiqi Xia[4†], Da Zhou[1†], Yingjie Ai[1], Linfeng Wu[2], Xiaoqing Zeng[1*], Shiyao Chen[1,2,3*]

Supplemental Digital Content

Supplementary materials and methods

Plasmid and siRNA transfection

Plasmids and siRNA were transfected using Lipo3000 Reagent (L3000015; Thermo Scientific). Each well of cells (5×10^5) was treated with 100 µL Opti-MEM Medium containing 2–3 µg plasmid or 50 pmol siRNA and 4–6 µL Lipo3000. The siRNA sequences are provided in Supplemental Table S2.

RNA extraction and real-time PCR

Total RNA was extracted from the liver tissues or cell lines using Trizol reagent (AN51L758; Life iLab, China) according to the manufacturer's instructions. RNA was reverse-transcribed using a cDNA Synthesis SuperMix for qPCR (11141ES60; Yeasen, China), and qPCR was performed on a StepOne Plus machine (ABI, USA). The mRNA levels of selected genes were calculated after normalization to β-actin by using the $2^{(-\Delta\Delta C(T))}$ method. Primer sequences are provided in Supplemental Table S3.

Immunoblotting

Mouse liver tissues and cells were homogenized and lysed in Protein Extraction Reagent (P0013; Beyotime Biotechnology, China). Protein lysates, in equivalent quantities, were subjected to separation via sodium dodecyl sulfate-polyacrylamide gel electrophoresis (SDS-PAGE) and subsequently transferred to polyvinylidene fluoride (PVDF) membranes (Millipore, USA). The antibodies are detailed in Supplemental Table S4. For normalization purposes, β-actin protein was utilized as a loading control.

Transcriptomic analyses

Total RNA was extracted from the liver tissues of *B4galt1*$^{f/f}$ and *B4galt1*$^{hep-/-}$ mice fed a chow diet or CDAHFD using the TRIzol Reagent. RNA purity and quantification were evaluated using the NanoDrop 2000 spectrophotometer (Thermo Scientific, USA). The libraries were constructed using VAHTS Universal V6 RNA-seq Library Prep Kit. The transcriptome sequencing was conducted by OE Biotech Co., Ltd. (Shanghai, China). Differential expression genes (DEGs) were identified using DESeq2 as those with a cutoff Benjamini-Hochberg adjusted *p* value of 0.05 and an absolute fold change of greater than 1.5 ($|Log_2FC| > 0.58$). Based on the hypergeometric distribution, GO enrichment analysis of DEGs were performed to screen the significant enriched term using R (v 3.2.0). Furthermore, the Gene Set Enrichment Analysis (GSEA) was conducted utilizing the GSEA software. The RNA-seq data are available in the GEO database under accession number GSE324244.

Measurements of iron concentration and lipid peroxidation

Levels of hepatic total iron were measured using a total iron assay kit (G4301; Servicebio, China) in accordance with the manufacturer's instruction. Levels of GSH, GSSG and MDA were quantified using glutathione and MDA assay kits (G4300/G4302, G4304; Servicebio, China). Lipid ROS was measured using the fluorescent probes BODIPY 581/591 C11 (L267; DOJINDO, China). Intracellular Fe^{2+} level was assessed using FerroOrange (L374; DOJINDO, China). AML12 cells were incubated with the kit reagent following the instruction at 37°C for 30 min in the incubator, and were imaged through a fluorescence microscope. The Image J software was used to evaluate fluorescence intensity.

Supplementary Figure Legends

Supplemental Fig 1. Correlation analysis between hepatic B4GALT1 expression and pathological scores. (A) Spearman correlation analysis of hepatic *B4GALT1* mRNA level with pathological scores in human MASLD liver samples (GSE174478, n=94). (B) Spearman correlation analysis of hepatic B4GALT1 level with pathological scores in patients with MASLD (n=30).

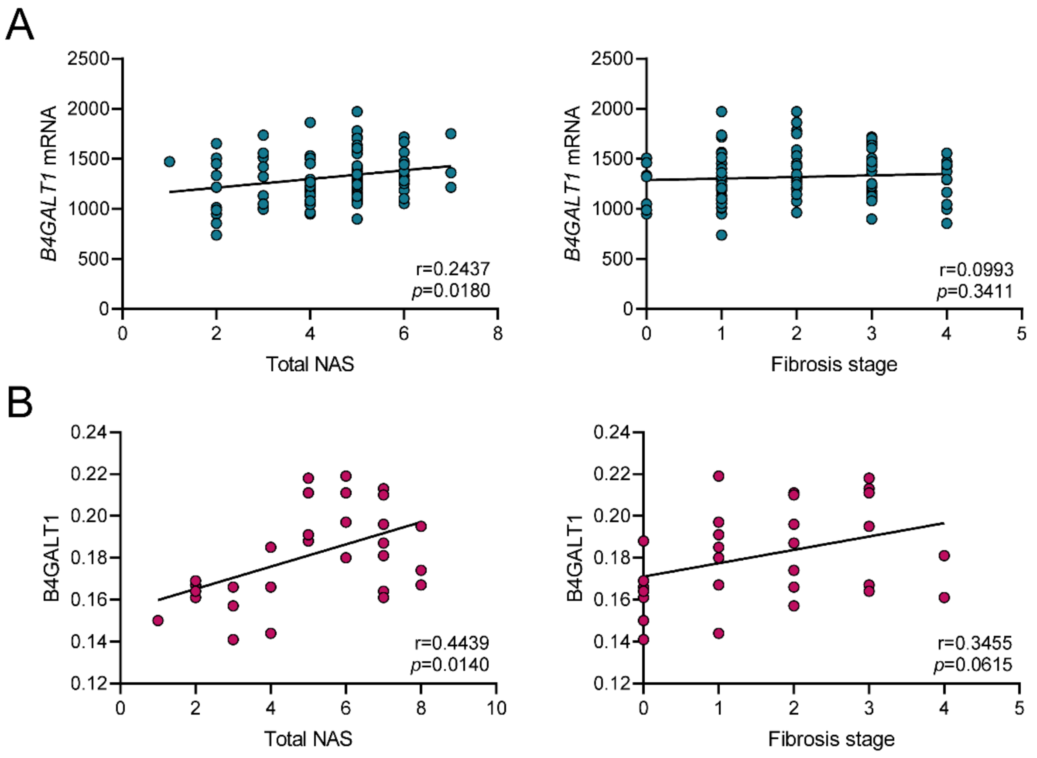

Supplemental Fig 2. Fibrosis-related genes and proteins expression levels in the liver of CDAHFD-induced MASLD mice. (A) Relative *Acta2, Col1a1,* and *Tgfb1* expression levels detected by qRT-PCR in CDAHFD-treated mice liver samples. (B) Relative α-SMA and MMP9 expression levels detected by western blot in CDAHFD-treated mice liver samples. Data are presented as mean ± SEM (**p <0.01, ***p <0.001, ns.: not significant; ANOVA test).

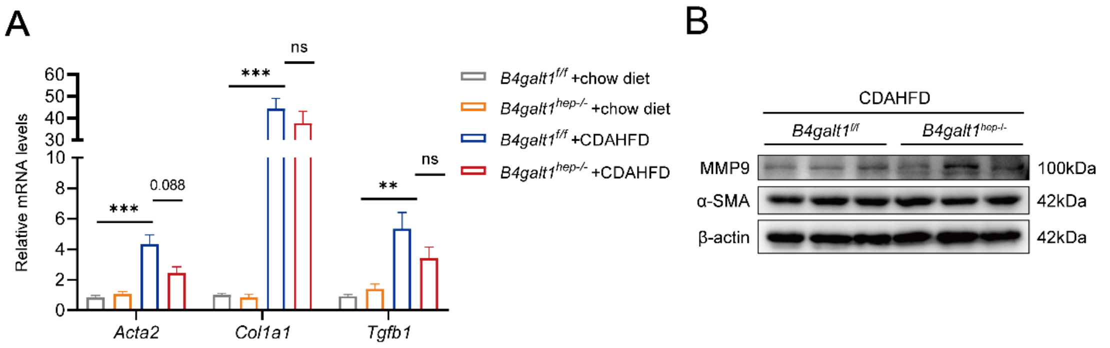

Supplemental Fig 3. B4GALT1 did not affect iron metabolism in the livers of CDAHFD-induced MASLD mice and in hepatocytes subjected to high-fat intervention. (A) Total iron concentration of liver tissues in normal and CDAHFD-treated mice. (B) Representative images of FerroOrange fluorescent staining in control, si*B4galt1* and *B4galt1* OE cells treated with or without FFA (0.5 mM) for 24 h. Scale bars, 50 µm. (C) Quantified mean fluorescence intensity of FerroOrange images (n=5/group). Data are presented as mean ± SEM (**p <0.01, ****p <0.0001, ns.: not significant; ANOVA test).

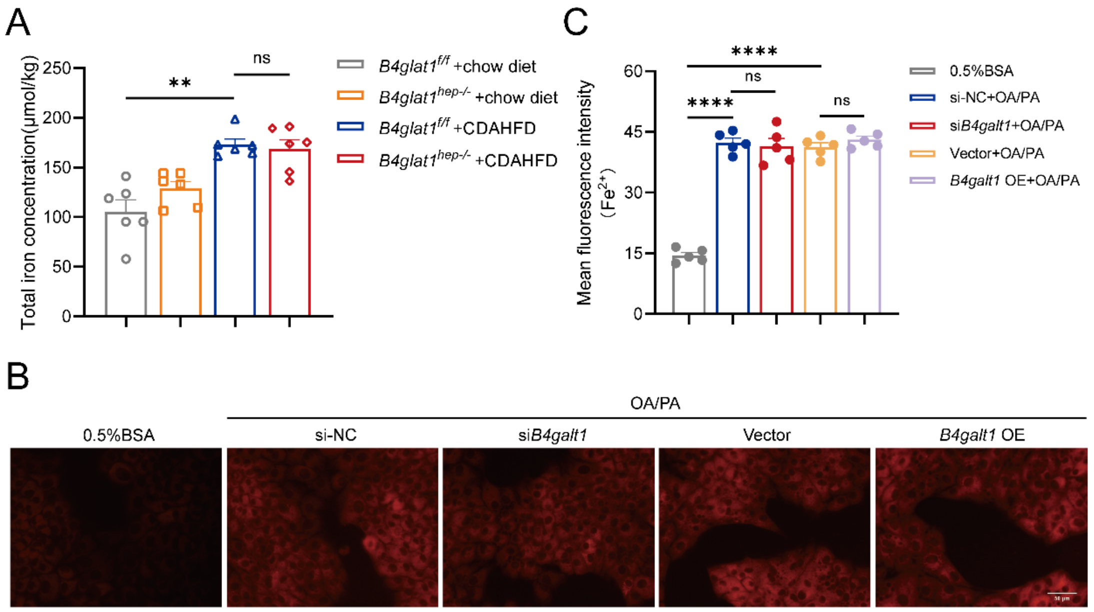

Supplemental Fig 4. Ferroptosis was involved in the protective effect of B4galt1 deficiency against lipid peroxidation in FFA-induced steatosis. (A–B) Level of MDA (A) and ratio of GSH to GSSG (B) in control and si*B4galt1* cells treated with DMSO or RSL3 (5 μM) for 24 h in combination with FFA (n=6/group). (C) Representative images of C11-BODIPY staining control and si*B4galt1* cells treated with DMSO or RSL3 (5 μM) for 24 h in combination with FFA. Scale bars, 50 μm. (D) Quantified mean fluorescence intensity of C11-BODIPY images (n=5/group). Data are presented as mean ± SEM (**p* <0.05, ***p* <0.01, ****p* <0.001, *****p* <0.0001; ANOVA test).

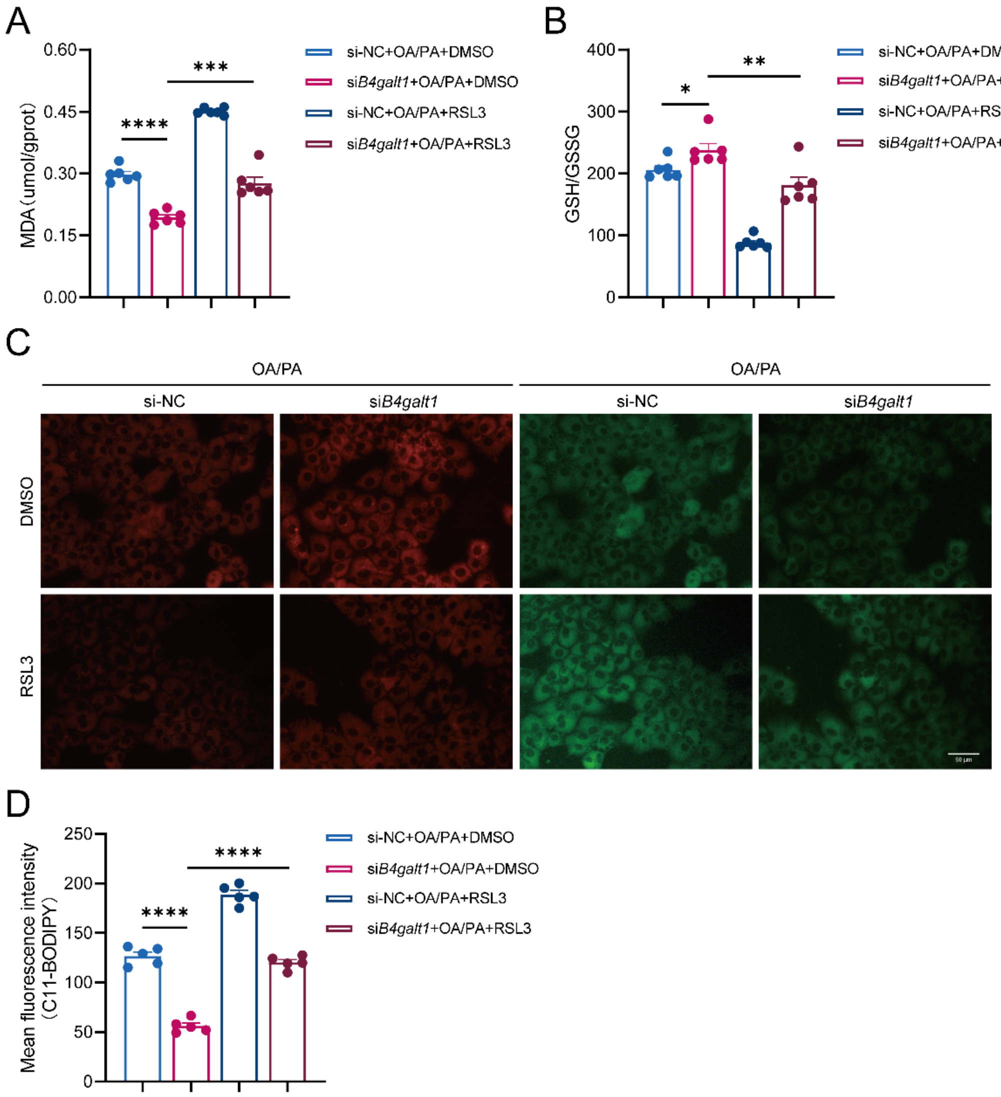

Supplemental Fig 5. Ferroptosis contributed to B4GALT1-aggravated lipid peroxidation in steatotic hepatocytes. (A–B) Level of MDA (A) and ratio of GSH to GSSG (B) in vector and *B4galt1*OE cells treated with DMSO or Fer-1 (10 µM) for 4 h in combination with FFA (n=6/group). (C) Representative images of C11-BODIPY staining vector and *B4galt1*OE cells treated with DMSO or Fer-1 (10 µM) for 4 h in combination with FFA. Scale bars, 50 µm. (D) Quantified mean fluorescence intensity of C11-BODIPY images (n=5/group). Data are presented as mean ± SEM (**p* <0.05, ***p* <0.01, ****p* <0.001, *****p* <0.0001; ANOVA test).

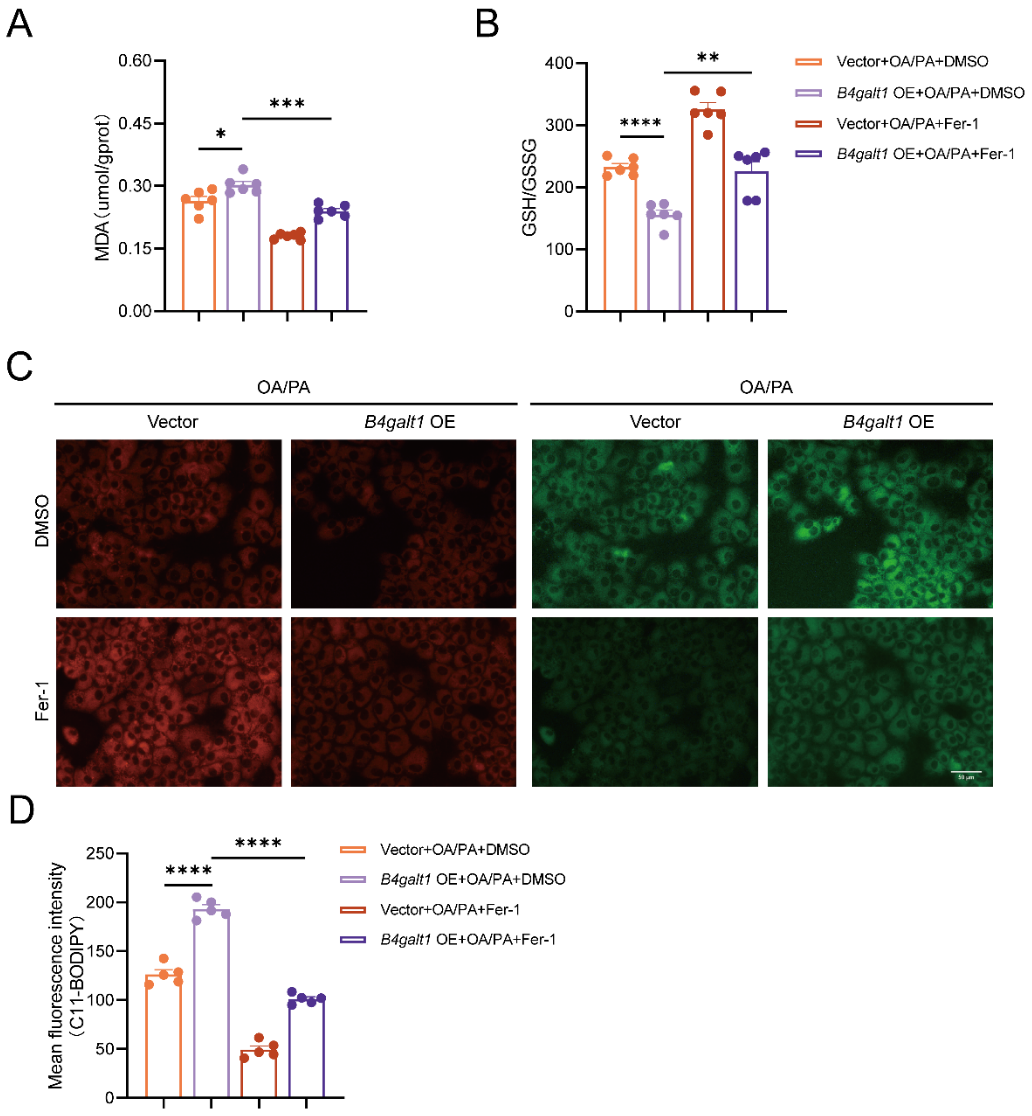

Supplemental Fig 6. B4GALT1 interacted with PPARγ in 293T cells. (A) Western blot analyses evaluating the interaction between B4GALT1 and PPARγ in 293T cells using exogenous Flag-B4GALT1 and Myc-PPARγ; empty vectors were used as control.

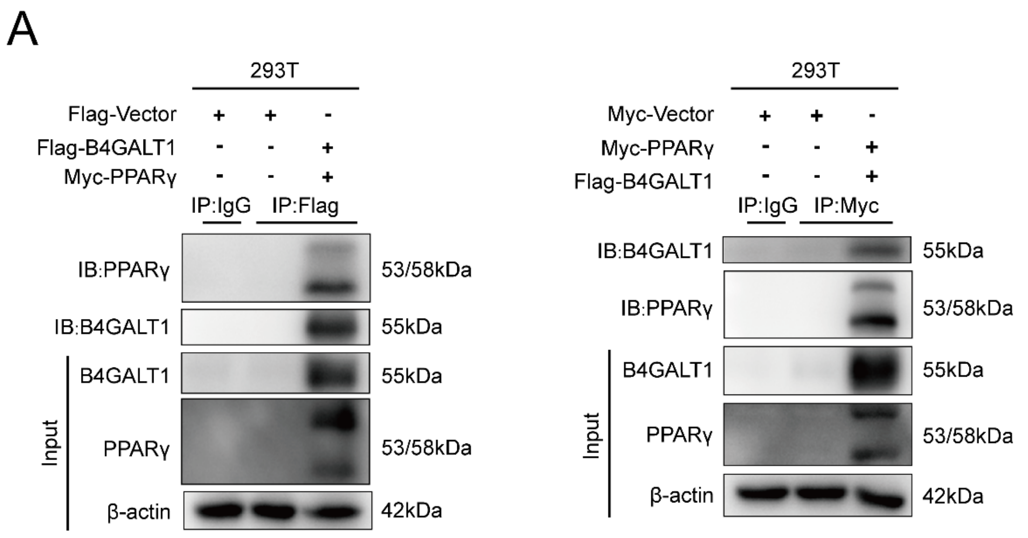

Supplemental Fig 7. B4GALT1 expression positively correlates with PPARγ targets across public MASLD transcriptomes. (A) Correlation heatmap of PPARγ target genes with B4galt1 in GSE285614 datasets from HFD-fed MASLD models. (B–C) Correlation heatmap of PPARγ target genes with B4galt1 in GSE196908 (12 w) and GSE246328 (24 w) datasets from GAN-fed MASLD models.

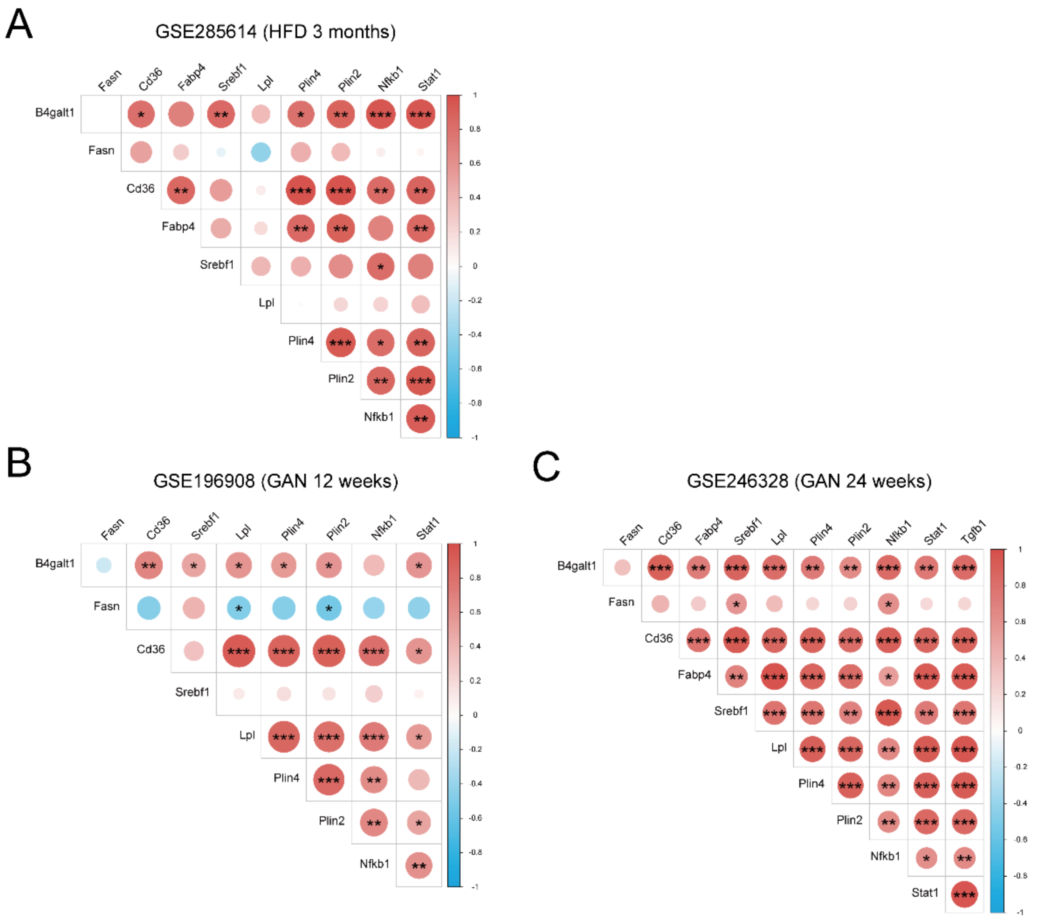

Supplemental Fig 8. PPARγ mRNA levels were significantly decreased in MASLD model upon B4GALT1 depletion. (A) Heatmap of PPARγ and its target genes expression in the livers of *B4galt1*$^{hep-/-}$ and *B4galt1*$^{flox/flox}$ mice. (B) Quantitative PCR analysis of *Pparγ* mRNA expression in liver samples from *B4galt1*$^{flox/flox}$ and *B4galt1*$^{hep-/-}$ mice fed a chow diet or CDAHFD. (C) Quantitative PCR analysis of *Pparγ* mRNA expression in AML12 cells transfected with *B4galt1* siRNA versus si-NC exposure to either 0.5% BSA or FFA. (**p* <0.05, ***p* <0.01, ****p* <0.001, *****p* <0.0001; ANOVA test).

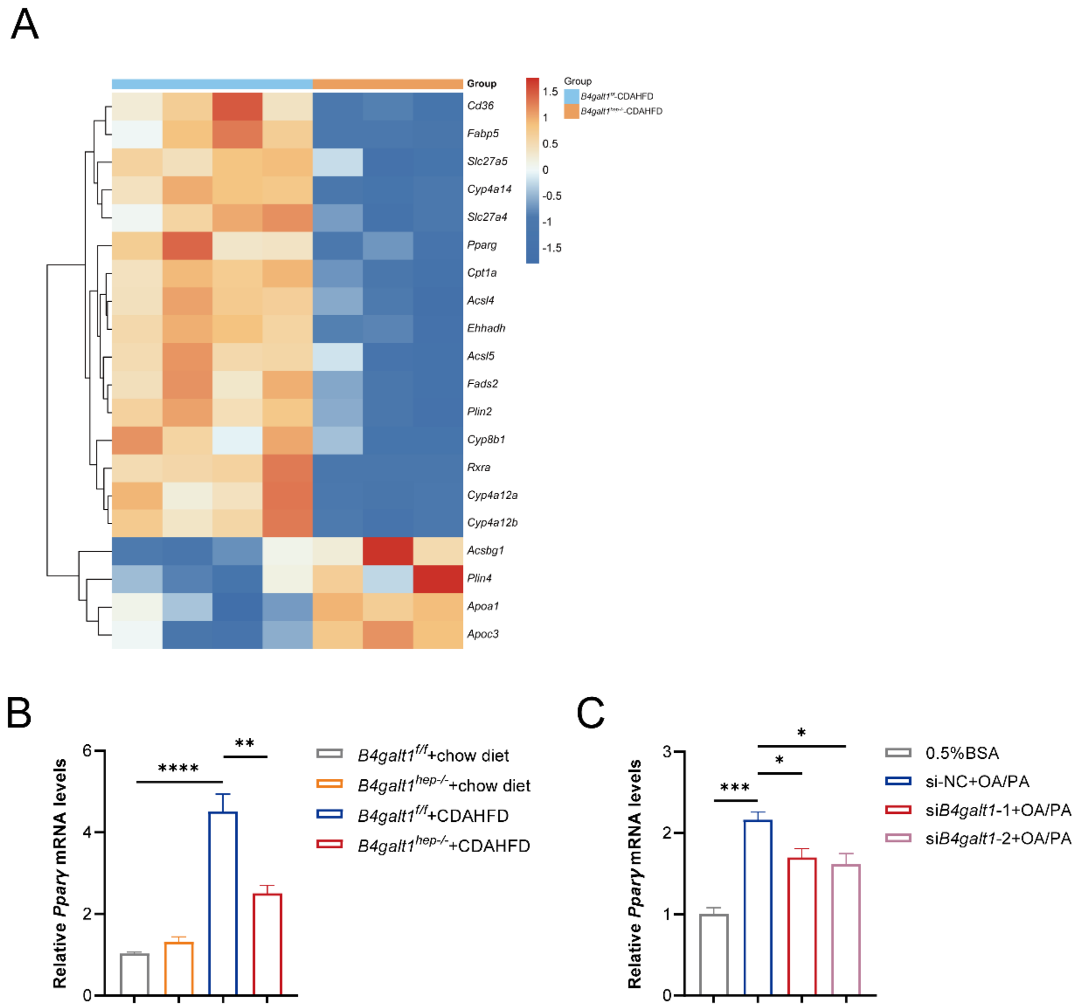

Supplemental Table 1. The characteristics of MASLD subjects

	NAS 1–2 (n=5)	NAS 3–4 (n=6)	MASH (n=19)	*p* value
Age (year)	55.6±18.7	41.0±13.4	45.5±12.9	0.055
Female	2 (40.0)	1 (16.7)	7 (36.8)	0.739
BMI	29.8±3.0	28.1±4.5	28.7±3.9	0.788
TC (mmol/L)	5.9±0.8	5.5±2.7	4.6±0.7	0.114
TG (mmol/L)	2.2±0.9	2.5±1.8	1.9±0.9	0.508
LDL (mmol/L)	3.8±1.1	3.6±2.9	2.7±0.8	0.251
HDL (mmol/L)	1.2±0.2	0.9±0.2	1.1±0.2	0.177
ALT (U/L)	81.2±16.7	84.3±31.5	89.0±12.9	0.960
AST (U/L)	48.2±9.3	40.8±7.2	55.1±5.5	0.399
ALB (g/L)	44.2±3.3	45.3±3.0	44.3±2.3	0.701
FBG (mmol/L)	5.5±0.3	5.0±0.7	5.5±1.3	0.576
CAP (dB/m)	339.0±37.1	323.7±23.4	319.7±56.4	0.740
E (kPa)	10.5±2.6	9.6±4.7	14.3±4.7	0.055
Hepatoprotectants	1 (20.0)	3 (50.0)	12 (63.2)	0.260

Note: Difference of age, BMI, TC, TG, HDL, LDL, ALT, AST, ALB, FBG, CAP, E among groups were determined by the ANOVA test. Difference of sex, drug taking among groups were determined by Fisher's exact test.

Supplemental Table 2. Sequences for *siRNAs*

Name	Sequences (5' to 3')	Supplier
B4galt1 siRNA#1	GGCAGGAGCATCTCAAATA	RiboBio
B4galt1 siRNA#2	CCATGCTGATTGACTTTAA	RiboBio

Supplemental Table 3. Primers for qRT-PCR

Name	Sequence	Supplier
m*B4galt1*-F	AGCAACTCGACTATGGCATCTACG	Sangon Biotech
m*B4galt1*-R	GGTCCACATCACTGAACACAAAGC	Sangon Biotech
m*Actb*-F	GATCATTGCTCCTCCTGAGC	Sangon Biotech
m*Actb*-R	ACTCCTGCTTGCTGATCCAC	Sangon Biotech
m*Acta2*-F	GTCCCAGACATCAGGGAGTAA	Sangon Biotech
m*Acta2*-R	TCGGATACTTCAGCGTCAGGA	Sangon Biotech
m*Col1a1*-F	GCTCCTCTTAGGGGCCACT	Sangon Biotech
m*Col1a1*-R	CCACGTCTCACCATTGGGG	Sangon Biotech
m*Tgfb1*-F	CCGCAACAACGCCATCTA	Sangon Biotech

m*Tgfb1*-R	ACTGCCGTACAACTCCAGTGAC	Sangon Biotech
m*Fasn*-F	AGGTGGTGATAGCCGGTATGT	Sangon Biotech
m*Fasn*-R	TGGGTAATCCATAGAGCCCAG	Sangon Biotech
m*Acaca*-F	TGACAGACTGATCGCAGAGAAAG	Sangon Biotech
m*Acaca*-R	TGGAGAGCCCCACACACA	Sangon Biotech
m*Srebf1*-F	GATGTGCGAACTGGACACAG	Sangon Biotech
m*Srebf1*-R	CATAGGGGGCGTCAAACAG	Sangon Biotech
m*Acsl4*-F	CTCACCATTATATTGCTGCCTGT	Sangon Biotech
m*Acsl4*-R	TCTCTTTGCCATAGCGTTTTTCT	Sangon Biotech
m*Acox1*-F	TAACTTCCTCACTCGAAGCCA	Sangon Biotech
m*Acox1*-R	AGTTCCATGACCCATCTCTGTC	Sangon Biotech
m*Acadm*-F	CAAGTTTGCCAGAGAGGAGATTATC	Sangon Biotech
m*Acadm*-R	AACGGGTACTCCCCGCTTT	Sangon Biotech
m*Cpt1a*-F	CTCCGCCTGAGCCATGAAG	Sangon Biotech
m*Cpt1a*-R	CACCAGTGATGATGCCATTCT	Sangon Biotech
m*Cpt2*-F	CAGCACAGCATCGTACCCA	Sangon Biotech
m*Cpt2*-R	TCCCAATGCCGTTCTCAAAAT	Sangon Biotech
m*Fabp1*-F	ATGAACTTCTCCGGCAAGTACC	Sangon Biotech
m*Fabp1*-R	CTGACACCCCCTTGATGTCC	Sangon Biotech
m*Cd36*-F	ATGGGCTGTGATCGGAACTG	Sangon Biotech
m*Cd36*-R	GTCTTCCCAATAAGCATGTCTCC	Sangon Biotech
m*Acsl5*-F	TCCTGACGTTTGGAACGGC	Sangon Biotech
m*Acsl5*-R	CTCCCTCAATCCCCACAGAC	Sangon Biotech
m*Tfr*-F	GTTTCCGCCATCTCAGTCATCAGG	Sangon Biotech
m*Tfr*-R	GGACTTCGCCGCAACACCAG	Sangon Biotech
m*Fth1*-F	TGCCATCAACCGCCAGATCAAC	Sangon Biotech
m*Fth1*-R	ATTCAGCCCGCTCTCCCAGTC	Sangon Biotech

Supplemental Table 4. Antibodies

Name	Species reativity	Application and dilution ratio	Supplier	Cat No.
Anti-B4GALT1	Human	WB (1:1000); IHC (1:200)	abcam	ab121326
Anti-B4GALT1	Human, Mouse	WB (1:1000); IHC (1:600)	LifeSpan BioSciences	LS-C368884
Anti-RCA I, Biotinylated		WB (1:2000); IHC (1:800)	Vector Laboratories	B-1085-1
Anti-F4/80	Mouse	IHC (1:400)	CST	70076
Anti-α-SMA	Human, Mouse	WB (1:1000)	ABclonal	A17910
Anti-α-SMA	Human, Mouse	IHC (1:18000)	abcam	ab124964
Anti- ACSL4	Human, Mouse	WB (1:5000)	Proteintech	22401-1-AP
Anti-SREBF1	Human, Mouse	WB (1:1000)	Abclonal	A25305

Anti-GPX4	Human, Mouse	WB (1:1000)	Abmart	T56959
Anti-xCT	Human, Mouse	WB (1:1000)	Affinity	DF12509
Anti-PPARγ	Human, Mouse	WB (1:2000); IHC (1:800)	Proteintech	16643-1-AP
Anti-PPARγ	Human, Mouse	ChIP (1:100)	CST	2443
Anti-FLAG Magnetic Beads		IP	MCE	HY-K0207
Anti-c-Myc Magnetic Bead		IP	MCE	HY-K0206
Protein A/G Magnetic Beads		IP; ChIP	MCE	HY-K0202
Anti-IgG		IP; ChIP	Proteintech	B900620
Anti-beta Actin	Human, Mouse	WB (1:5000)	HUABIO	ET1702-67

Supplemental Table 5. Chemicals and reagents

Name	Supplier	Cat no.
DMEM/F-12	Gibco	11320-033
ITS (100x)	Sigma-Aldrich	I3146
Dexamethasone	Sigma-Aldrich	D4902
Oleic acid	MACKLIN	O815203
Palmitic acid	MACKLIN	P815432
Lipofectamine™ 3000	invitrogen	L3000001
Protease inhibitor cocktail	ABclonal	RM02916
Fer-1	Selleck	S7243
RSL3	Selleck	S8155
Pioglitazone	Selleck	S2590
GW9662	Selleck	S2915
Tunicamycin	Selleck	S7894
Cycloheximide	MCE	HY-12320
16% Formaldehyde	CST	12606
